# ASO Author Reflections: Vascular Pancreatic Surgery Worldwide: A Call to Strengthen Rescue and Standardize Care

**DOI:** 10.1245/s10434-025-18134-7

**Published:** 2025-08-19

**Authors:** Camila Hidalgo Salinas, Pascale Tinguely, Dimitri A. Raptis, Giuseppe Kito Fusai

**Affiliations:** 1 Nuffield Department of Primary Care Health Sciences, Centre for Evidence-Based Medicine, University of Oxford, Radcliffe Observatory Quarter, Oxford, UK; 2 Department for Continuing Education, University of Oxford, Oxford, UK; 3 Department of HPB Surgery and Liver Transplant, Royal Free Hospital NHS Foundation Trust, London, UK; 4 Organ Transplant Center of Excellence, King Faisal Specialist Hospital and Research Centre, Riyadh, Saudi Arabia

## Past

Pancreas surgery involving vascular resection, particularly arterial involvement, has historically been considered high risk and typically confined to high-volume, specialized centers.^1^ While these institutions have reported favorable outcomes, these experiences may not reflect global practice.^2^ Until now, prospective data capturing consecutive patients undergoing vascular pancreatic surgery across diverse healthcare systems have been lacking, limiting our understanding of real-world outcomes worldwide.^3,4^

## Present

In this prospective, international snapshot study by PancreasGroup.org, we analyzed 3926 patients undergoing pancreatic surgery across 317 centers worldwide, with 565 undergoing concomitant vascular resections across 202 centers.^5^ This included 444 patients with venous resection and 121 patients with arterial resection and reconstruction (alone or combined). Remarkably, postoperative complication rates were comparable across countries with a varying Human Development Index (HDI), suggesting that surgical technique can be standardized globally regardless of resource constrains; however, this apparent equity masks critical disparities in rescue care. The 8.8-fold increase in mortality when arterial resection and reconstruction was complicated by clinically relevant pancreatic fistula underscores that institutional capacity for complication management, not surgical technique alone, determines patient survival. Additionally, only 36% of patients undergoing arterial resection received neoadjuvant chemotherapy, representing a significant gap between guideline recommendations and clinical practice worldwide.

## Future

Our findings reframe our understanding of global vascular pancreatic surgery outcomes. Success requires more than technical expertise; it demands institutional preparedness for complications and equitable access to multimodal therapy. While comparable complication rates demonstrate that surgical standardization is achievable globally, the profound impact of failure-to-rescue (FTR) events highlights that healthcare system capacity remains a determining factor. Future research must focus on identifying modifiable hospital-level factors that influence FTR, particularly in resource constrained settings. International collaboration is essential to promote high-quality pancreatic cancer care worldwide.
